# Trauma systems in Asian countries: challenges and recommendations

**DOI:** 10.1186/s13054-024-04838-x

**Published:** 2024-02-16

**Authors:** Dinesh Bagaria, Amila S. Ratnayake, Aireen Madrid, Tamara J. Worlton

**Affiliations:** 1 Department of Trauma, All India Institute of Medical Sciences, New Delhi, India; 2 Military Hospital, Colombo, Sri Lanka; 3 Uniformed Services University of the Health Sciences, Bethesda, MD USA; 4 Department of Trauma, Philippine General Hospital, Manila, Philippines

**Keywords:** Trauma systems, Emergency care, LMIC, Asia, Trauma system development

## Abstract

**Introduction:**

Trauma burden is one of the leading causes of young human life and economic loss in low- and middle-income countries. Improved emergency and trauma care systems may save up to 2 million lives in these countries.

**Method:**

This is a comprehensive expert opinion participated by 4 experts analyzing 6 Asian countries compiling the most pressing trauma care issues in Asia as well as goal directed solutions for uplifting of trauma care in these countries.

**Result:**

Lack of legislation, stable funding under a dedicated lead agency is a major deterrent to development and sustainment of trauma systems in most Asian countries. While advocating trauma, critical care as a specialty is a key event in the system establishment, Trauma specialized training is challenging in low resource settings and can be circumvented by regional cooperation in creating trauma specialized academic centers of excellence. Trauma quality improvement process is integral to the system maturity but acquisition and analysis of quality data through trauma specific registries is the least developed in the Asian setting.

## Introduction

The Sustainable Development Goals (SDG) 2030 established by the United Nations (UN) envisioned “all people enjoy health, justice and prosperity [1]. It is critical that no one is left behind.” Specifically, the most disadvantaged, marginalized, and hard-to-reach populations should be lifted up by the SDGs. Healthy people are the foundation for healthy economies. According to a recent research based on the World Health Organization (WHO)’s Projecting the Economic Cost of Ill-Health (EPIC) model, for LMIC and low-income countries (LIC), injury accounts for the majority of annual loss of gross domestic product (GDP) due to surgical disease [2]. The loss of GDP for all surgical diseases in countries of all income statuses is a staggering 20.7 trillion USD and is projected to continue to rise. Disease burdens that can be managed by emergency and trauma services amount to 30% of the global disease burden, yet over 70% of the world’s population cannot access safe, timely and affordable emergency surgical and critical care when they need it. By developing emergency and trauma care systems to the level of high-income countries, the number of lives that can be saved rises to 2 million [3–5].

The context of global trauma differs considerably according to geographical, epidemiological, socioeconomic, political, and health systems attributes. There are varying system configurations and maturation to adapt to those diverse perspectives and system strains [6–8].

Within those diverse perspectives, trauma systems are composed of multiple integrated components and agents with varying inter-relationships, invisible boundaries, and system strains. Systems evolve and mature within these complexities in varying configurations and best practices [9].

Four experts, three representing Asia and one from the United States with multiple on site experiences of working with trauma systems in Asia, endeavored to compile current status of regional trauma systems and draw recommendations. All three Asian experts were actively involved in trauma system development in their own countries and principal investigators of an ongoing research project in Asian Collaboration for Trauma, an organization with 17 affiliated organizations and 288 fellows aiming to advocate for trauma system development in Asia.

## Contemporary status of trauma systems in Asia

In Asia, the capacity to cope with the rapidly growing injury problem needs strengthening. The top ten countries based on population density are all in Southeast Asia where approximately 8% of the world’s population lives on 3% of the world’s land mass [10]. If India is included, this region accounts for 25% of the world’s population.World Bank defines those with incomes of more than $12,615 as high-income countries. By 2015 more than 95% of people in the Asia region lived in middle-income economies. Countries in the region that have moved up include the newly-industrialized economies of Hong Kong, China; the Republic of Korea; Singapore; and Taipei, China [11].

According to the 2023 update to achieving the SDGs, the density of doctors in Southern Asia and South-Eastern Asia ranks the lowest in the world after Sub-Saharan Africa [12]. Additionally, many of the countries in this region have their population spread across multiple islands, creating unique challenges in trauma care. It is well established that morbidity and mortality from road traffic injury is disproportionately high in low-and-middle income countries (LMIC), but little is discussed about violence. Looking at the global terrorism index, six countries in the region have an index equal to or greater than the United States which has a well-known domestic terrorism issue. This would likely be regionally higher; however, many countries incidents are unreported [13].

Although components of a comprehensive trauma system are visible in Asia, they vary in configuration and stages of development (Table 1). Traditionally, trauma education is the most cost effective and first focus when starting system organization. In most national trauma educational endeavors, professional organizations provide leadership in the establishment and dissemination of trauma courses. Most of the countries rely on the American College of Surgeon (ACS)’s Committee on Trauma’s Advanced Trauma Life Support (ATLS) as the standard, but the prohibitive cost is a major drawback to establish and sustain the ATLS program [14]. To circumvent this barrier, many countries develop and distribute low-cost training programs from international or local organizations such as the National Trauma Management Course (NTMC) [15]. Trauma training in medical school is not prioritized in Asia and there are huge disparities in advanced training in trauma and critical care fellowships [16]. Many countries embark on advanced trauma definitive care courses, though cultural taboos in using animal models or human cadavers and the prohibitive cost of manikins and other training materials hinder widespread dissemination. Participating in trauma programs at regional educational hubs in Singapore and the Philippines is cost-effective and should be regionally expanded. Regional professional collaboration in trauma education and capacity building is a positive trend in Asia. For example, Alfred Health from Australia has funded and mentored trauma development projects in multiple Asian countries including India, Sri Lanka, and China [17–19].

**Table 1:** Summary of current status and recommendations for Asian Trauma system development

System domain	Status in mature trauma systems	Current status in Asia	Developmental recommendation
National Lead Agency	Governance and oversight are provided by a federal (or national) lead agency with statutory/legislative powers to provide the continuum of trauma care, from prevention, through pre-hospital, hospital to rehabilitation. The lead agency to be informed and advised by a national or regional multistakeholder expert advisory group	Lacking appropriate authority, staffing, and funding if exists at all	∙ Governments must identify and empower a national organization to lead the trauma development process
Prehospital	∙ National or regional creation of a lead Emergency Medical Services (EMS) agency to formulate policies, laws, rules and regulations for pre-hospital trauma care ∙ EMS should be activated on universal access number, integrated into inclusive network of healthcare facilities, and connected ∙ There are command and control centers equipped with state-of-the-art information technology to take int patients to correct pre alerted health care facility according to the patient needs by using a predefined triage tool ∙ EMS is resourced with well-equipped land (and air where resources allow air) ambulances and trained licensed crew ∙ Maintain national standards and subjected to continuous process improvement with tracking of key performance indicators	EMS is mature in urban settings but lacks national standards	∙ A national lead agency should create, certify, and maintain national standards for professional EMS ∙ EMS should coordinate with regional trauma hubs to deliver the right patient to the right hospital ∙ Funding and mechanism to resupply EMS materials used to care for trauma patients
Facility based care	Availability of specialized level trauma centers (Level 1/Major Trauma Center), designated and verified by national accreditation body, authorized by a statutory document, with resources (human & material) available 24/7 to manage the most severely injured patients	∙ Lack of trauma trained professionals ∙ Lack of regional trauma hubs	∙ Establish trauma training programs ∙ Designate and fund hospitals to become the regional trauma facility
Rehabilitation	∙ Availability of in-hospital, specialist (e.g., neurotrauma) and community rehabilitation services integrated within a trauma system or network, enacted by law/trauma center accreditation, with oversight by the rehabilitation director ∙ Rehabilitation services and outcome assessment data should be captured by trauma registry and subjected to continuous performance improvement	Injury rehabilitation takes place within the hospital	∙ Create multidisciplinary teams dedicated to the injured patient ∙ Regional trauma hospitals should have affiliated but separate injury rehabilitation centers
Trauma quality improvement	National trauma registry with minimal comprehensive data set should be instigated. Data should be captured by trained professionals to include continuum of patient care from prehospital through definitive care to post discharge community care and rehabilitation/recovery outcomes. The registry will facilitate KPI benchmarking,	National trauma statistics, data acquisition, data analysis, data specialists are lacking	∙ Support a single national trauma registry ∙ Provide mechanism for hospitals to report data that does not rely on healthcare workers ∙ Use information to advocate for injury prevention and legislation development

Emergency Medicine (EM) physicians are needed allies in trauma care, education, and advocacy, particularly in bridging the prehospital to in-hospital training, however it is a relatively new specialty globally and although most countries in Asia have training programs, the density of EM physicians is still low [19]. In recent times, emergency medical services (EMS) system development is seen in parallel to establishing emergency medicine as postgraduate specialty in multiple Asian countries including Thailand, Sri Lanka, and Philippines. Strengthening of prehospital care through training of community-based providers and use of staffed community ambulances has been estimated to cost less than 100 USD per disability-adjusted life year averted or per life saved and has been shown to reduce mortality by 25–50% in some LMIC settings [20].

EMS have developed in varying configurations to match the diverse geographical, epidemiological, and socio-economic landscape prevailing in Asia, however, except in Sri Lanka [21] (SUWASERIYA foundation—http://www.1990.lk/) and Thailand [22] (National Institute for Emergency Medicine NIEM—https://www.niems.go.th/1/Home/Main) there is no statute passed in parliament for a lead agency to govern or a national policy to streamline the developmental process and secure funding to sustain the effort. There are significant gaps in coordination and communication between the multiple organizations participating in EMS and the receiving healthcare facilities for efficient use of healthcare resources.

Most Asian countries established the EMS system as a pilot program and then later expanded as a nationwide endeavor [23].There are varying EMS personnel providing on scene and en route care including emergency medical technicians, paramedics and EM physicians, however national standards in EMS training and education are lacking in most countries. There is no national body to govern EMS training standards and education except in Thailand. Most countries have established national toll-free telephone number to increase access to EMS with commendable coverage. Thailand EMS utilizes community participation effectively with structured community bystander training programs (community emergency volunteers—CEVs) to increase coverage that can be emulated in other Asian countries [24].

An inclusive system of trauma specific hospitals (regionalized trauma care) similar to the Global North [25–27] are still to be established in the Asian region. Though most of the investigated countries have well-established public health systems under the Ministry of Health (MoH) with established referral pathways, most trauma care is done by generalists without specific training or necessarily interest in trauma care. Hospitals are not incentivized to invest in robust trauma care as universal healthcare compensation inadequately reimburses for operative and nonoperative trauma.

National trauma registry, data analysis, research, and trauma quality improvement are in their infancy in Asia except for Thailand [28]. Although there are documented efforts to launch national trauma registries in the region, they have been stalled due to sustained funding or rely too heavily on already burdened healthcare workers to do data entry. Policy for trauma and emergency care development drafted in Thailand, Vietnam and Sri Lanka has only partially been implemented due to lack of political commitment and advocacy leading to funds diverting to other competing national healthcare issues. In the Philippines, trauma surgeons are key advocates for injury prevention with the national laws regarding helmet use, seat belt use, and bans on distracted driving were drafted by trauma surgeons [29]. These laws were based on data from other countries and a national trauma registry is needed to data drive culturally specific injury prevention strategies. The WHO is working with Member States to strengthen national injury-related data system development, including injury surveillance, health information systems and vital registration [4, 30, 31].

## Challenges in trauma system development and recommendations

### Clearly identified and appropriately funded national lead agency

Lack of legislation, stable funding under a dedicated lead agency is a major deterrent to development and sustainment of trauma systems in most Asian countries [32]. When successful implementation of trauma development programs has been completed, there is a strong political mandate with legislation and an identified lead agency to oversee the process [33].

Public awareness and enthusiasm with stakeholder engagement is vital in driving political commitment to sustain the trauma system development process. Lack of federal/national leadership and an identified and appropriately funded lead agency results in poor coordination and collaboration of public, private, and other agencies responsible for emergency and trauma care services. Most successful trauma projects adhere to locally grown contextually applicable methods in trauma network development [33, 34].

### Advocacy for the specialty of trauma

Trauma is not an attractive or even financially viable discipline for general surgeons in the already strained Asian health sector. To develop the workforce, emphasis and remuneration for mentors, infrastructure, and teaching material needs to be prioritized. With this, widespread dissemination of advanced trauma courses can be accomplished as culturally appropriate in Asian countries, with a new emphasis on not just initial certification, but re-certification as well.

### National registries and quality improvement

Trauma registries and quality improvement processes are the least developed out of the areas studied and affected by funding, lack of infrastructure, and technology [35, 36]. Trauma data and statistics abstracted through generic health data, audits, and surveillance is documented in most Asian countries however lacks granularity when hospital based, fails to account for prolonged pre-hospital transfers and other causes of death prior to admission to hospital. A national lead agency dedicated to data collection, audit, and research will be the first step in the journey towards national trauma data banks curated by trauma registries with published annual reports to facilitate policy making and resource allocation [6, 37]. The WHO minimal dataset and other low-cost resources will facilitate the visualization of essential data needed for trauma system governance and financing [38].

### National standards for pre-hospital care

Considering the spectrum of geography, population distribution, disease burden, and socio-economic landscapes that prevail in Asia, it is not possible to recommend a unique trauma system that fits all settings. Establishing the generic component of the regionalized, inclusive trauma system or contextually appropriate substitute with integration and continuous process improvement is the way forward to efficient trauma system in Asia. EMS is critical to bring injured patients to well-resourced centers capable of providing standard care in a timely manner. It is essential to connect EMS, their dispatch center, and health facility emergency teams to garner better outcomes from EMS operations. Initial funding and expertise from developed nations is seen as a practical solution with a win–win situation to both participants [39, 40].

Public and private partnerships should assist in sustaining the services in the context of limited public/government funding.

Widespread stakeholder training is essential to gain full potential of the system. Locally curated low-cost training model adhering to the international core principals works best for Asia. Wider community participation with structured bystander training and community volunteers’ programs is a cost-effective model. Many Asian countries have adopted and adapted the ACS Stop the Bleed course.

### Create national trauma training hubs

Trauma training networks centered on regional high profile academic centers will facilitate advanced trauma training for surgeons and residents to circumvent high investment needed in launching and sustenance of those proprietary training models. Pre-identified tertiary care, university, or national hospitals should be upgraded with available resources as trauma centers under the leadership of national trauma experts. These hubs should be funded to train trauma fellows. A unique solution in the Philippines brings remote provincial surgeons to Manila to be trauma trained funded by their home hospital. These trauma surgeons then owe years of service to the provincial hospital, thus spreading trauma advocacy and education nationally and counteracting migration of physicians to large cities.

Task sharing and task shifting is an option where there is considerable scarcity of trained specialized human resources. Training non specialist doctors on specific procedural knowledge and skills where expert healthcare staff is lacking, and high patient burden has proven cost effective [41–43].

### Prioritize injury prevention and rehabilitation

Prevention of injuries and safety promotion need to be institutionalized under the nationally identified lead agency and not depend on individual surgeons. An injury unit should be established to implement injury prevention and coordinate with other sectors. Asian countries need to enact comprehensive legislation on injury prevention and ensure better enforcement of these laws. Acute trauma care and services at national and local levels need to be strengthened to ensure effective care and rehabilitation services. Most important is to move rehabilitation from the expensive and inefficient in hospital system to dedicated rehabilitation centers. These actions will call for the involvement of the private sector well as civil society [44].

## Conclusion

### Key messages

- Lack of legislation, stable funding under a dedicated lead agency is a major deterrent to development and sustainment of trauma systems in most Asian countries.
- Advocating trauma critical care as a specialty and trauma critical care specialty colleges will fuel the trauma system policy development, securing stable funding, implementation, establishing trauma network, and maturity through data collection, analysis and research.
- A national lead agency dedicated to data collection, audit, and research will be the first step in the journey towards national trauma data banks curated by trauma registries with published annual reports to facilitate policy making and resource allocation.

Neglecting the development of emergency and trauma care systems is too costly in terms of both human life and GDP. Varying geography, diseases, and socio-political landscapes in Asia need contextually appropriate system development. By focusing on the contained recommendations (Table 1) delete, Asian countries can systematically improve national trauma care, reduce the drain of injury on GDP, and save countless lives.

## Declarations

### Disclaimers

The opinions or assertions contained herein are the private ones of the author/speaker and are not to be construed as official or reflecting the views of the Department of Defense, the Uniformed Services University of the Health Sciences or any other agency of the U.S. Government.
